# Competitive Interactions of Ligands and Macromolecular Crowders with Maltose Binding Protein

**DOI:** 10.1371/journal.pone.0074969

**Published:** 2013-10-04

**Authors:** Andrew C. Miklos, Matthew Sumpter, Huan-Xiang Zhou

**Affiliations:** 1 Institute of Molecular Biophysics, Florida State University, Tallahassee, Florida, United States of America; 2 Department of Physics, Florida State University, Tallahassee, Florida, United States of America; New England BioLabs, United States of America

## Abstract

Cellular signaling involves a cascade of recognition events occurring in a complex environment with high concentrations of proteins, polysaccharides, and other macromolecules. The influence of macromolecular crowders on protein binding affinity through hard-core repulsion is well studied, and possible contributions of protein-crowder soft attraction have been implicated recently. Here we present direct evidence for weak association of maltose binding protein (MBP) with a polysaccharide crowder Ficoll, and that this association effectively competes with the binding of the natural ligand, maltose. Titration data over wide ranges of maltose and Ficoll concentrations fit well with a three-state competitive binding model. Broadening of MBP ^1^H­^15^N TROSY spectra by the addition of Ficoll indicates weak protein-crowder association, and subsequent recovery of sharp NMR peaks upon addition of maltose indicates that the interactions of the crowder and the ligand with MBP are competitive. We hypothesize that, in the *Escherichia coli* periplasm, the competitive interactions of polysaccharides and maltose with MBP could allow MBP to shuttle between the peptidoglycan attached to the outer membrane and the ATP-binding cassette transporter in the inner membrane.

## Introduction

Cellular signaling processes involve a cascade of recognition events that lead from an external stimulus to a cellular response. Each recognition event is the binding of a protein with a partner that can range from a small-molecule ligand to a macromolecular complex. The binding occurs in a complex environment containing high concentrations of proteins, polysaccharides, and other macromolecules [1]. Binary interactions in the cascade are often studied separately in dilute solution. It is now recognized that the crowded cellular environment can significantly influence the equilibria of protein binding [2], [3]. While *in vivo* studies in principle could capture environmental effects, such studies are confounded by many factors, including spatial and temporal variations in macromolecular concentrations in a cell, variations from cell to cell, as well as off-pathway processes [4]. The use of macromolecular crowding agents in *in vitro* studies allows for quantitative characterization of protein binding processes in conditions mimicking the cellular environment, leading to a more complete understanding of recognition and signaling processes inside the cell. Here we report the effects of a polysaccharide crowder on the binding affinity of maltose binding protein (MBP) for its natural ligand maltose.

**Figure 1:**
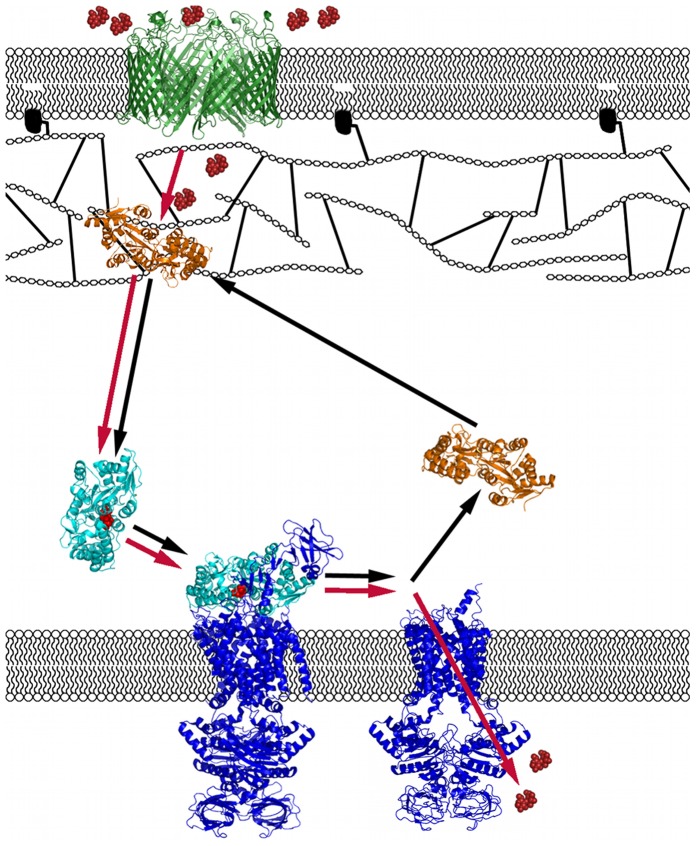
Illustration of the maltose transport system in the periplasm. The components are shown in different colors: maltose (red), maltoporin (green), apo MBP (orange), maltose-bound MBP (cyan), and ABC transporter (blue). Red arrows indicate the flow of maltose from the extracellular space into the cytoplasm, while black arrows indicate the signaling cycle for MBP. Peptidoglycan is represented with hexagons for the glycan chains, and lines for oligopeptide linkers between glycan chains.

**Figure 2:**
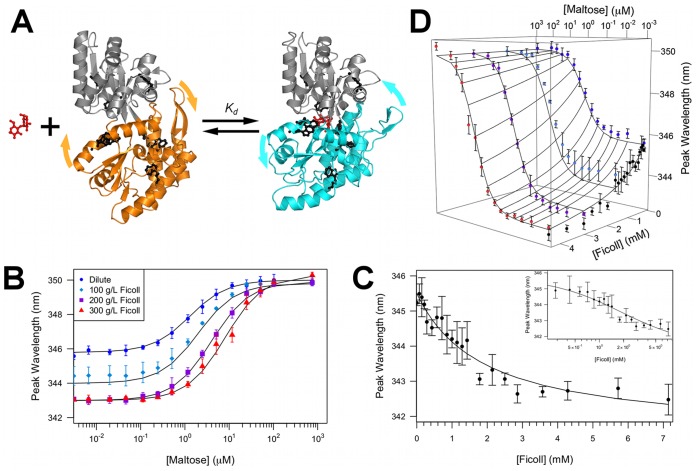
Titrations of maltose or Ficoll into MBP, monitored by the peak wavelength of tryptophan fluorescence. (A) The maltose-MBP binding equilibrium. Apo and maltose-bound MBP are shown with the N-terminal lobes aligned and in gray, and the C-terminal lobes in orange and cyan, respectively, to illustrate the closure of the lobes upon ligand binding. The 8 tryptophan residues are shown as black sticks. (B) Maltose titration at fixed concentrations of Ficoll. Data at 0 maltose are shown on the vertical axis. (C) Ficoll titration in the absence of maltose. Inset: same data with Ficoll concentration in log scale. (D) Global fit of all the titration data to the three-state competitive binding model. Symbols represent experimental data (error bars are S.E.M. of 3 or 4 independent measurements), while curves represent the fits. Data at 0 maltose are shown at 10^−3^ µM to be included on the log scale. All experiments were performed at 37 °C in 100 mM sodium phosphate buffer (pH 7.2).

MBP is a member of the family of periplasmic binding proteins, involved in nutrient uptake and chemotaxis for *Escherichia coli* cells [5]. These proteins must transport their ligands across the periplasmic space and interact with the ATP binding cassette (ABC) transporters in the inner membrane to relay signals to the cytoplasm of *E. coli* cells (Figure 1). Since its discovery in 1971 [6], MBP’s interactions with upstream and downstream partners have been extensively studied in dilute solution. An open structure for the apo form and a closed structure for the maltose-bound form have been determined (Figures 1 & 2A) [7], [8]. There is evidence suggesting that the degree of binding cleft opening is a determinant of ligand affinity [9], [10]. Recently, a semi-closed structure has been proposed for the apo form as a minor population and its role in the ligand binding process has been implicated [11]–[13]. Conformational plasticity has also been shown in the interaction of MBP with the ABC transporter [14].

Despite this extensive knowledge of MBP from studies in dilute solution, many unanswered questions remain about how MBP functions within cells. It is known that a significant fraction of MBP is immobilized in the periplasm in the absence of maltose but becomes mobile when maltose is present [15], but it is unclear where in the space between the outer and inner membranes apo MBP is localized. Additionally, it is uncertain how the periplasmic environment, which is more crowded than the cytoplasm [16], influences the binding properties of MBP. Overall crowding in the periplasm is further complicated by peptidoglycan (PG), a large, highly crosslinked macromolecule comprising amino acids and polysaccharides, which is attached to the outer membrane (Figure 1) [17].

Studies on the effects of macromolecular crowding have focused on hard-core repulsion between proteins and crowders, which is universal and expected to favor compact conformations [2], [3]. Computational studies have revealed that repulsive crowders can indeed increase the closed populations of ligand-binding proteins, thus favoring the bound state over the unbound state [18]–[21]. Experimental studies using either Ficoll [21] or a small molecule crowder [22] have confirmed an increase in closed populations, suggesting the role of protein-crowder hard-core repulsion. The data in the latter study were analyzed to show that the crowder resulted in a significant increase in the ligand binding affinity. Meanwhile, other recent studies have implicated roles of protein-crowder soft attraction in modulating protein binding or folding stability [23]–[31]. Characterizing the effects of the soft attraction will be of utmost importance in formulating a complete picture of the crowded environment inside cells.

In the present study of MBP-maltose binding, we chose the polysaccharide Ficoll 70 as the crowder. This choice was motivated in part by the consideration that, relative to MBP, the much larger size of Ficoll 70 would render the hard-core repulsion contribution minimal [20], therefore providing an opportunity to isolate the soft attraction contribution. In contrast to an increase in MBP-maltose binding affinity expected from a hard-core repulsion contribution, a significant decrease in binding affinity (amounting to 6.7-fold increase in apparent dissociation constant) was observed in the presence of 300 g/l Ficoll. Direct evidence for protein-crowder weak association was presented by significant broadening of MBP ^1^H­^15^N TROSY spectra by the addition of Ficoll. Moreover, maltose and Ficoll appear to interact competitively with MBP. Sharp NMR peaks were recovered upon adding a saturating amount of maltose. In addition, titration data over wide ranges of maltose and Ficoll concentrations fit well with a three-state competitive binding model. The weak, competitive attraction by polysaccharides may be important for MBP’s cellular function. Specifically, we hypothesize that this weak attraction enables apo MBP to be localized within the PG mesh, where it is primed to receive maltose arriving through maltoporin. Binding of incoming maltose could then release MBP, allowing it to freely diffuse to the ABC transporter and hand over the maltose for translocation into the cytoplasm (Figure 1).

## Results

### Competitive Binding of Maltose and Ficoll to MBP

MBP contains 8 tryptophan residues, dispersed around the ligand-binding cleft and throughout the whole protein (Figure 2A). A significant red-shift in the peak wavelength of the tryptophan fluorescence was observed upon titration with maltose (Figure 2B). This red-shift was used to monitor the binding equilibrium. Fitting of the titration curve in dilute solution to a two-state binding model (Figure 2A) yielded a dissociation constant of 1.2 ± 0.1 µM, consistent with previous studies [32].

In the presence of increasing amounts of Ficoll 70, the titration curves gradually shifted toward higher maltose concentration (Figure 2B). The apparent dissociation constant (*K*_d;app_) increased by 6.7-fold to 8 ± 1 µM at 300 g/L Ficoll (Table 1). The decrease in maltose binding affinity of MBP (as shown by the *K*_d;app_ increase) by Ficoll is in distinct contrast to an increase in ATP binding affinity of adenylate kinase, attributed to hard-core repulsion, by the small molecule osmolyte, trimethyl amine N-oxide [22].

**Table 1:** Parameters from Fitting of Titration Data.

Two-State Model			
Titrant	*K*_d;app_ or 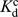 (µM)	λ_f_ or λ_P_(nm)	λ_b_ or λ_C•P_(nm)
Maltose ([C]_T_ = 0)^a^	**1.2 ± 0.1**	**345.8 ± 0.1**	**350.0 ± 0.1**
Maltose ([C]_T_ = 100 g/L)	1.9 ± 0.3	344.0 ± 0.1	349.8 ± 0.2
Maltose ([C]_T_ = 200 g/L)	3.9 ± 0.2	343.0 ± 0.1	349.9 ± 0.1
Maltose ([C]_T_ = 300 g/L)	8 ± 1	343.0 ± 0.1	350.3 ± 0.1
Ficoll ([L]_T_ = 0)^a^	**2000 ± 500**	345.6 ± 0.1	**341.5 ± 0.4**
**Three-State Model**			
	***K*** **_d_ (µM)** 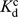 **(µM)**	**λ_P_ (nm)**	**λ_b_ (nm)** **λ_C•P_ (nm)**
Maltose or Ficoll	1.3 ± 0.1	345.8 ± 0.1	350.0 ± 0.1
	1500 ± 200		341.8 ± 0.2

a Bold entries from these two isolated titration experiments can be compared with the corresponding parameters in the global fit to the three-state model.

The peak fluorescence wavelength of apo MBP showed a discernable blue-shift upon addition of Ficoll (Figure 2B). The extent of the blue-shift initially increased with increasing Ficoll concentration, but interestingly, became nearly constant above 200 g/L (or, equivalently, 2.9 mM) Ficoll, as if reaching saturation of a binding isotherm. This observation prompted us to collect fluorescence data on apo MBP upon titration with Ficoll (Figure 2C). The data could be fitted to an MBP-Ficoll binding equilibrium with a dissociation constant of 2.0 ± 0.5 mM (or 140 g/l), indicating only weak association.

On the other hand, when MBP was saturated with maltose, the peak fluorescence wavelength was constant when increasing amounts of Ficoll were added (Figure 2B). This observation suggests that maltose-bound MBP cannot further bind Ficoll. In other words, maltose and Ficoll appear to bind competitively to MBP, but the binding affinities differ by three orders of magnitude.

To further support the notion of competitive binding, we fitted all the titration data, covering maltose concentrations from 0 to 753 µM and Ficoll concentrations from 0 to 4.3 mM, to a three-state model, in which MBP could bind either to maltose or Ficoll, but not both at the same time.
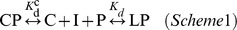



This fit (Figure 2D) yielded values for the five parameters matching (to within fitting errors; Table 1) those from the two-state fitting of two isolated titration experiments, either with maltose titrated in without Ficoll or vice versa. Control experiments with sucrose (Figure S1) show that the effects of the monomeric subunit of Ficoll on MBP-maltose binding are distinctly different from those of Ficoll, and are incompatible with the competitive binding model.

### Direct Evidence for MBP-Ficoll Weak Association from NMR Spectroscopy

NMR spectroscopy is uniquely able to directly probe weak association between a protein and a macromolecular crowder. The weak association can result in broadening of resonances (even to the point beyond detection) in ^1^H-^15^N HSQC or TROSY spectra of the protein, owing to the large size of the protein-crowder complex [33]. However, complex formation under weak association necessitates a high concentration of the crowder, and the resulting high macroviscosity itself could lead to broadening of resonances. Under conditions of mild resonance broadening, the effects of weak association and macroviscosity can be distinguished by measuring NMR relaxation data [33].

Our titration data suggested that not only does Ficoll weakly bind to MBP, but also Ficoll binding is competitive against maltose binding. The competitive binding afforded an opportunity to distinguish the effects of weak association and macroviscosity. If the addition of Ficoll results in broadening of apo MBP resonances and the broadening is due to MBP-Ficoll weak association, we expected that subsequent addition of a saturating amount of maltose, without affecting solution viscosity, would break the MBP-Ficoll complex and thus bring back sharp NMR peaks (of maltose-bound MBP).

**Figure 3:**
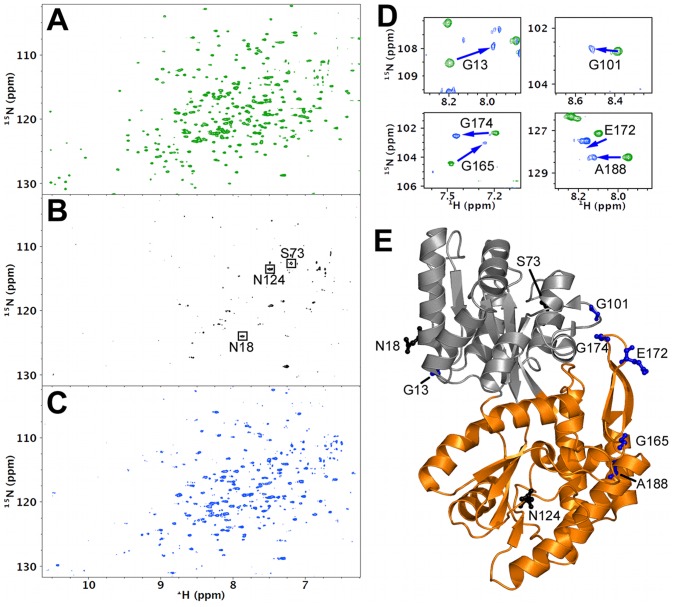
^1^H-^15^N TROSY spectra of MBP. Solution conditions were: (A) buffer only; (B) 200 g/L Ficoll; and (C) 200 g/L Ficoll with 1 mM maltose. Three assigned resonances are shown in (B). Data were collected at 37 °C in 10 mM sodium phosphate buffer (pH 7.2). (D) Enlarged views showing the movements of six assigned resonances when solution conditions were changed from (A), green, to (C), blue. (E) Presentation of the residues indicated in (B) and (D) on the structure of apo MBP.

We collected ^1^H-^15^N TROSY spectra on samples of ^15^N-enriched MBP in dilute solution, 200 g/L Ficoll, and 200 g/L Ficoll with 1 mM maltose (Figure 3A–D). Resonances observed in the dilute solution spectrum (Figure 3A) matched well with previously published spectra of apo MBP [34]. Addition of 200 g/L Ficoll resulted in the disappearance of over 90% of the NMR peaks (Figure 3B), indicating severe broadening. Some of the remaining observable resonances could be easily matched to the known assignments of apo MBP, and the assigned residues (including Asn18, Ser73, and Asn124; Figure 3B) correspond to highly mobile residues in turns and loops (Figure 3E), showing that the protein was still intact but rotationally impeded due to weak association with Ficoll and/or high macroviscosity.

To ascertain that weak association was responsible for the loss of NMR signal, 1 mM maltose was added to the crowded sample. The titration data above show that this concentration is saturating for maltose binding to MBP in 200 g/L Ficoll (Figure 2B,D). Upon addition of maltose, the lost peaks were recovered (Figure 3C). Among the recovered resonances, those that were easily assigned by ^1^H and ^15^N chemical shifts alone (> 0.2 weighted ppm from other possible resonances) and shifted appreciably (> 0.2 weighted ppm) from the counterparts in the apo MBP spectrum matched the previous assignments of ligand-bound MBP [34]. The assignment of these resonances (including Gly13, Gly101, Gly165, Glu172, Gly174, and Ala188; Figure 3D,E) demonstrated that maltose outcompeted Ficoll for binding with MBP.

## Discussion

### Competitive Inhibition of MBP-Maltose Binding by MBP-Ficoll Weak Association

The present study presented direct evidence for weak association between MBP and the polysaccharide crowder Ficoll, and showed that this weak association is competitive with the binding of the natural ligand maltose. Titration with Ficoll, as monitored by MBP fluorescence peak wavelength, exhibited saturation, and yielded a weak, mM dissociation constant (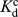). Moreover, broadening of ^1^H-^15^N TROSY spectra directly indicated weak association of MBP with Ficoll.

The competitive nature of the interactions of Ficoll and maltose with MBP was also shown by both the fluorescence data and the NMR data. While the apparent peak wavelength for the maltose-free species exhibited significant change with varying Ficoll concentrations, the counterpart for the maltose-bound species remained constant. Our interpretation is that the maltose-free species actually consisted of two MBP populations, one free of Ficoll and one associated with Ficoll; the ratio of these two populations changed with the Ficoll concentration. On the other hand, the maltose-bound species was a single population, as MBP could not bind to maltose and Ficoll at the same time. Again, the NMR spectroscopy provided direct evidence for the competition of Ficoll and maltose for binding to MBP. When a saturating amount of maltose was added to sample with 200 g/L Ficoll, sharp NMR peaks were recovered, indicating that now the maltose-bound MBP was free of association with Ficoll.

The weak, competitive association with Ficoll inevitably resulted in a decrease in maltose binding affinity. The three-state competitive binding model predicted a linear dependence of the apparent ligand dissociation constant on the Ficoll concentration (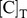): 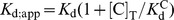. Using the values of parameters from fitting two isolated titration experiments, each involving two components (i.e., MBP along with either maltose or Ficoll as titrant), we essentially were able to reproduce the titration curves with maltose as the titrant and Ficoll at various fixed concentrations (Table 1 and Figure 2D). This observation provides quantitative support for the conclusion that Ficoll weakly associates with MBP and thereby competitively inhibits maltose binding.

For MBP, it has been demonstrated via mutagenesis that reducing the binding cleft opening results in increased affinity for maltose [9]. Based on this observation, we can conclude that the decreased maltose affinity in the presence of Ficoll cannot be attributed to protein-crowder hard-core repulsion. The repulsion would favor closed conformations over open conformations, and thus lead to increased affinity for maltose. Using a computational approach known as postprocessing [19], [20], we predict that hard-core repulsion by 200 g/l Ficoll would result in a 6% increase in the population ratio of the closed to open conformations, as well as the same very modest decrease in the apparent dissociation constant for maltose. The predicted effect of hard-core repulsion is thus opposite to the measured results for Ficoll, and in any event, is so small as to likely fall within experimental errors.

The decrease in maltose binding affinity along with MBP-Ficoll weak association observed here is reminiscent of the effects of synthetic antigen binders (sABs) that preferentially bind to the open conformations of MBP [10]. However, compared to the sABs, the association of Ficoll with MBP is much weaker, and the selectivity for the open conformations over the closed conformations is likely much lower. The weak association of MBP with Ficoll is likely due to nonspecific, soft attraction, in line with other recent studies of crowding effects [23]–[31]. A preference for the open conformations can arise because they present larger surface areas with which MBP can interact. It has been predicted that the destabilizing effect of the soft attraction increases as the temperature is lowered [23], [29]. Our measurements of the effects of Ficoll on the MBP-maltose binding affinity at lower temperatures (not shown) are consistent with this predicted trend. More studies are needed to fully characterize the nature of the MBP-Ficoll interactions.

Crowded solutions present unique challenges to biophysical characterizations of proteins. In particular, NMR spectra of proteins upon addition of crowders and inside cells can be broadened to the point beyond detection (present study and refs. [33], [35]). Here it was by taking advantage of the competition for interactions that we were able to use maltose to free MBP from weak association with Ficoll. A similar strategy can perhaps be useful for studying other proteins under crowding.

### General Role of Protein-Crowder Soft Attraction

Weak association between proteins and macromolecular crowders is likely to be a general phenomenon both in crowded solutions and inside cells. Under those conditions, as noted above, broadening of NMR peaks is commonly observed; like the MBP-Ficoll system studied here, protein-crowder weak association was shown to be the main cause for the broadening in some other cases as well [33]. Our preliminary data (Figures S2 & S3) indicate that other macromolecular crowders (dextran and bovine serum albumin) also decrease the maltose binding affinity, again implicating protein-crowder weak association. We therefore infer that periplasmic proteins in *E. coli* may generally have an inhibitory effect on MBP-maltose binding.

Interactions between test proteins and macromolecular crowders are expected to consist of hard-core repulsion and longer-ranged weak attraction. The balance between these two components will dictate the net effect of a crowder on the thermodynamic properties of a test protein [29]. While hard-core repulsion has been the focus of many studies on macromolecular crowding [2], [3], there is growing appreciation for the potential contributions of soft attraction [23]–[31].

The role of protein-crowder weak attraction cannot be overlooked when considering a full picture of the crowded environment inside cells. As shown in the present study, a crowder that weakly associates with a protein, even when the affinity is three orders of magnitude lower than that of the protein’s natural ligand, can notably influence the amount of ligand required to effect a change in ligand-bound populations. This type of inhibition from crowded cellular components could contribute to the noise observed in many signaling processes [4]. Although Ficoll is a synthetic polymer crowder and is absent in cells, other macromolecular analogues present inside the periplasm may have similar effects on the signaling properties of MBP.

While the protein-crowder weak association observed here results in an inhibitory effect on ligand binding, it can have a beneficial effect on cellular signaling under specific circumstances. As we argue below, weak association could steer signaling proteins to regions of the cell where they are more receptive to extracellular inputs.

### Possible Implications for MBP in Cellular Signaling

In the periplasm, 28% of MBP is immobilized under maltose-poor conditions, but essentially all of the MBP becomes mobile under maltose-rich conditions [15]. This observation is strikingly similar to our NMR data showing that apo MBP weakly associates with the polysaccharide crowder Ficoll and maltose-bound MBP is free from this association.

The interaction partner(s) of apo MBP that causes this reversible loss of mobility in the periplasm is uncertain. Evidence of an interaction between the outer membrane protein maltoporin and MBP could explain the immobilization, but 0.5 M maltose was insufficient to disrupt the interaction *in vitro* [36], [37]. Although an MBP-maltoporin interaction cannot be discounted, we alternatively suggest that the immobilization of apo MBP is due to weak association with the PG mesh (Figure 1). We single out PG for several reasons. First, PG accounts for most of the macromolecular mass in the periplasm, constituting 2.5% of the dry weight of the cell [38], compared to a combined 2% for all the periplasmic proteins [39]. Second, evidence for MBP-PG interaction has been presented by a pull-down assay in which MBP was mixed with isolated PG sacculi and subjected to ultracentrifugation [40]. 20% of the protein was found in the PG pellet, suggesting nonspecific binding of MBP with PG. Third, immunoelectron microscopy has shown that the MBP of a Gram-positive bacterium is localized mostly in the PG layer [41]. In this case, the bacterium contains no outer membrane (and hence no outer membrane proteins with which to associate), yet MBP is still localized within the cell wall, which is primarily composed of PG.

Much in the same way that MBP weakly associates with Ficoll (ready to dissociate upon binding maltose), we hypothesize that apo MBP weakly associates with PG. In this way, apo MBP can be localized near the outer membrane, and is primed to receive maltose diffusing in from the extracellular medium through maltoporin (Figure 1). When exposed to the ligand, MBP can detach from the PG layer, and freely diffuse towards the inner membrane. There, maltose-bound MBP can then interact with the ABC transporter and chemotactic signal transducer to transport nutrients to the cytoplasm and initiate chemotaxis. Other periplasmic binding proteins may exploit a similar mechanism involving weak association with PG for shuttling between the outer and the inner membranes.

## Materials and Methods

### Protein Expression and Purification

The pMal-c2x vector system (New England Biolabs) was mutated to replace the C-terminal linker with a double stop codon, resulting in an ampicillin-resistant plasmid that expresses cytoplasmic, full-length MBP. Natural abundance proteins were expressed and purified using the method of Charlton et al. [42], with the following changes: LB media was used in place of Spectra 9 media, lysis was achieved by microfluidization, anion exchange buffers comprised 20 mM Tris, 1 mM PMSF, 2 mM DTT, and 0.1 mM EDTA, pH 7.4 with or without 1 M NaCl, and dialysis and subsequent size exclusion chromatography were performed using 100 mM sodium phosphate buffer (pH 7.2).

Expression of ^15^N-enriched MBP was performed using ^15^N minimal media as described previously [26], with purification performed as above with two alterations. The buffer used for dialysis and size exclusion chromatography was 10 mM phosphate (pH 7.2), owing to salt concentration constraints imposed by CryoProbe systems [43]. Additionally, the protein was subjected to a denaturation, desalting, and refolding process as described in Tang et al. [11] to ensure no residual bound oligosaccharides remained.

### Titration Monitored by Tryptophan Fluorescence

Experiments were performed on a Cary 300 Fluorimeter using 200 nM MBP in 100 mM sodium phosphate buffer (pH 7.2) at 37 °C. Maltose titrations were performed by addition of small volumes of high concentration maltose solutions to the MBP sample, with a total volume change of <3% over each titration. Crowder titrations were conducted with multiple samples, as addition of crowding agent to concentrations reaching 300 g/L was not feasible in the same sample without large volume and MBP concentration changes.

Data were analyzed using two different models: a two-state binding model (Figure 2A), and a three-state competitive binding model (Scheme 1). For the two-state model, the maltose titration data at a fixed Ficoll concentration were fitted to the following equation:
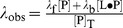
(1)with



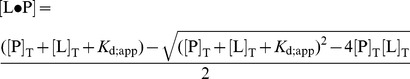
(2)

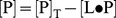
(3)where λ_obs_, λ_f_, λ_b_ were observed, ligand-free, and ligand-bound fluorescence peak wavelengths; [P]_T_, [L]_T_ were total protein and ligand concentrations; and *K*_d;app_ was the apparent dissociation constant. The three fitting parameters λ_f_, λ_b_, and *K*_d;app_ were free to change at different Ficoll concentrations. The Ficoll titration data in the absence of maltose were fitted to a similar two-state binding model. The free and bound peak wavelengths were now denoted as λ_P_ and λ_C•P_; and the dissociation constant as 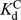. Because the MBP-Ficoll association was weak (i.e., 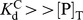 used in the experiments), the counterpart of eq 2 for 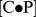 could be simplified to
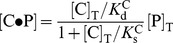
(4)where [C]T was the total crowder concentration.

All the maltose and Ficoll titration data were also globally fitted to the three-state model. For our case of protein-Ficoll weak binding (i.e., 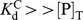), the predicted peak wavelength could be couched in the form of eq 1, but with [P], λ_f_, and *K*_d;app_ reinterpreted. Now, [P] represented the sum of the two ligand-free species, i.e., the free protein P and the protein-crowder complex.


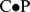
; the ligand-free wavelength was
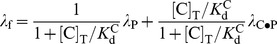
(5)and the apparent dissociation constant was
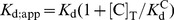
(6)where K_d_ denoted the dissociation constant in the absence of Ficoll. The fitting involved five global parameters: λ_P_, λ_C•P_, λ_b_, K_d_ and 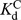.

### NMR Spectroscopy

Samples for NMR spectroscopy were 0.1 mM MBP, 10 mM sodium phosphate buffer (pH 7.2), with 12% D_2_O and 0.2% sodium azide. Crowded samples contained 200 g/L Ficoll 70, and maltose­saturated samples were generated by addition of 1 M maltose to 200 g/L Ficoll samples for a final maltose concentration of 1 mM. ^1^H­^15^N TROSY spectra were collected at 37 °C on a Bruker 700 MHz spectrometer equipped with a triple­resonance CryoProbe.

## Supporting Information

Figure S1 **MBP fluorescence changes upon sucrose titration in the presence and absence of saturating maltose.** A linear response to sucrose was observed; the lack of a binding transition indicates that there is no apparent 1∶1 binding to MBP even at the highest sucrose concentrations measured (∼1.5 M without maltose and ∼0.9 M with maltose). Experiments performed at 37 °C using 200 nM MBP in 100 mM sodium phosphate buffer (pH 7.2).
(TIFF)

Figure S2 **Effect on MBP-maltose binding by 300 g/L dextran-40.** Tryptophan fluorescence spectra measured at 25 °C using 200 nM MBP in 100 mM sodium phosphate buffer (pH 7.2). Apparent two-state fitting yields a *K*_d_;app value of 6 ± 1 µM.
(TIFF)

Figure S3 **MBP-maltose binding in buffer and in crowded solutions.** Titrations of maltose into 20 nM NTT-labeled MBP were monitored by Microscale Thermophoresis. Solutions contained: (A) buffer only; (B) 200 g/L Ficoll; and (C) 100 g/L BSA. *K*_d,app_ values are 3 ± 1 µM, 6 ± 2 µM, and 14 ± 4 µM, respectively. All experiments performed on a Monolith NT.115 at 25 °C in 100 mM sodium phosphate buffer (pH 7.2) as described by Wienken CJ *et al.* (Nature Communications 1: doi:10.1038/ncomms1093).
(TIFF)
